# Stridor in Bilateral Medial Medullary Infarction: A Case Report, Literature Review, and Pathophysiologic Insights Into a Rare Presentation of an Uncommon Stroke

**DOI:** 10.1155/crnm/5348210

**Published:** 2026-01-12

**Authors:** Marvic Joseph S. Amoranto, Ramon Carlos Miguel L. Alemany, Norman N. Mendoza

**Affiliations:** ^1^ Department of Neurology, Institute of the Neurological Sciences, The Medical City, Pasig, Philippines, themedicalcity.com; ^2^ Ear-Nose-Throat, Head and Neck Surgery Institute, The Medical City, Pasig, Philippines, themedicalcity.com

**Keywords:** anterior spinal artery, bilateral medial medullary infarction, nucleus ambiguus, stridor, vertebral artery

## Abstract

**Background:**

Upper airway obstruction secondary to bilateral vocal cord paralysis is not a known classic presentation of bilateral medial medullary infarction (BMMI). This may potentially confound the diagnostic approach, particularly when coexisting with bulbar symptoms and quadriplegia. Prompt recognition is essential for timely and appropriate airway management and subsequent treatment.

**Case Presentation:**

A 74‐year‐old female presented with a three‐week stepwise progression of asymmetric quadriparesis, slurred speech, and a prominent biphasic stridor. Flexible fiberoptic laryngoscopy revealed bilateral vocal cord palsy in the median–paramedian position, and an emergency tracheostomy was performed. Magnetic resonance imaging (MRI) of the brain revealed the characteristic “heart shaped” diffusion‐weighted imaging (DWI) pattern of BMMI, while magnetic resonance angiography (MRA) exhibited absent flow‐related signals in the right vertebral artery. Secondary stroke prevention with clopidogrel was started. However, the patient developed severe pneumonia with massive pleural effusion and expired on the sixth day of hospitalization due to Type 1 respiratory failure.

**Conclusion:**

Bilateral vocal cord paralysis may occur in BMMI, and recognizing this rare association is crucial for timely diagnosis and treatment. The intricate neurovascular anatomy of the medulla may find insight into the rarity of this association.

## 1. Background

Medial medullary infarction (MMI) is a rare stroke subtype, accounting for only 0.5%–1.5% of all strokes, whereas bilateral medial medullary infarction (BMMI) is even rarer [1, 2]. Bilateral involvement of the medial medulla produces a spectrum of neurologic deficits that classically include quadriparesis, dysarthria, nystagmus, and hypoglossal palsy [3–5]. The diverse and complex clinical manifestations of BMMI can pose a diagnostic challenge, particularly for practitioners less familiar with uncommon features of acute cerebrovascular disease. Prompt recognition is critical, as involvement of the nuclear groups governing central respiratory control within this region of the brain places patients at risk for fatal respiratory failure [5–7]. Early detection facilitates timely and appropriate airway management, including tracheostomy when necessary.

We present a case of BMMI that, beyond the classical neurological deficits, also manifested with signs of upper airway obstruction due to bilateral vocal cord paralysis, which is an exceptionally underreported clinical feature of this kind of stroke with only one prior laryngoscopically confirmed case in our literature review [8].

## 2. Case Presentation

A 74‐year‐old female with uncontrolled hypertension and insulin‐requiring Type 2 diabetes mellitus initially developed sudden‐onset right upper extremity weakness. This progressed to involve both lower limbs, followed by slurred speech, noisy breathing, and quadriparesis. The symptoms evolved in a stepwise pattern for 3 weeks. On the 19th day of symptom progression, a noncontrast cranial computed tomography (CT) scan was performed and found to be unremarkable for an acute vascular event. On the 24th day, the patient presented to the emergency department (ED) with a blood pressure (BP) of 150/90 mmHg and a heart rate of 108 beats per minute. She exhibited a prominent biphasic stridor yet remained mildly tachypneic, with a respiratory rate of 22 breaths per minute and an oxygen saturation of 98% at room air. She was awake, coherent, and able to promptly follow commands but dysarthric and was unable to protrude her tongue. She had incomplete quadriplegia with a Medical Research Council (MRC) scale for muscle strength of 0/5 on the right upper and lower limbs, 3/5 on the left upper limb, and 2/5 on the left lower limb, with hyperreflexia and bilateral Babinski signs.

**Figure 1:**
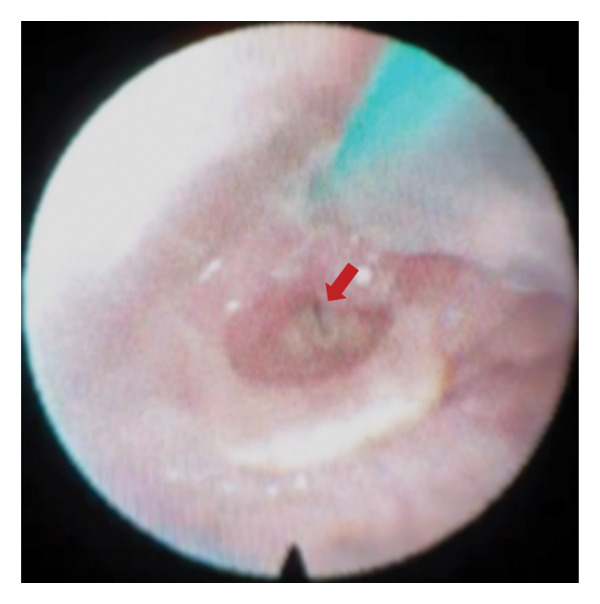
Flexible fiberoptic laryngoscopy showing bilateral paralysis of the vocal cords in the median–paramedian position (red arrow).

Random blood sugar was 180 mg/dL with an HbA1c of 9.8% (84 mmol/mol). Electrocardiogram (ECG) showed sinus tachycardia with fusion complexes. Chest radiography exhibited pneumonia in the left lung, with an elevated procalcitonin level of 3.95 ng/mL, and antibiotics were started. Flexible fiberoptic laryngoscopy revealed paralysis of the bilateral vocal cords in the median–paramedian position (Figure 1). Given the neurological manifestations of the patient, a central type of vocal cord paralysis was considered. Upon referral to neurology service, a clinical diagnosis of BMMI was made, and an emergency tracheostomy was performed to secure a patent airway. Thereafter, the patient was admitted in the acute stroke unit.

**Figure 2:**
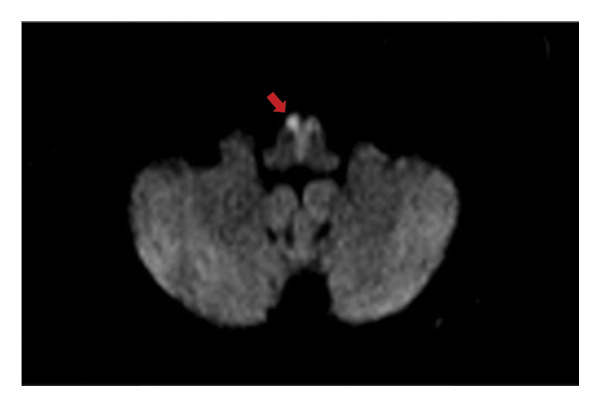
Area of restricted diffusion at the bilateral ventromedial aspects of the medulla resembling a “heart‐shaped” appearance (red arrow).

**Figure 3:**
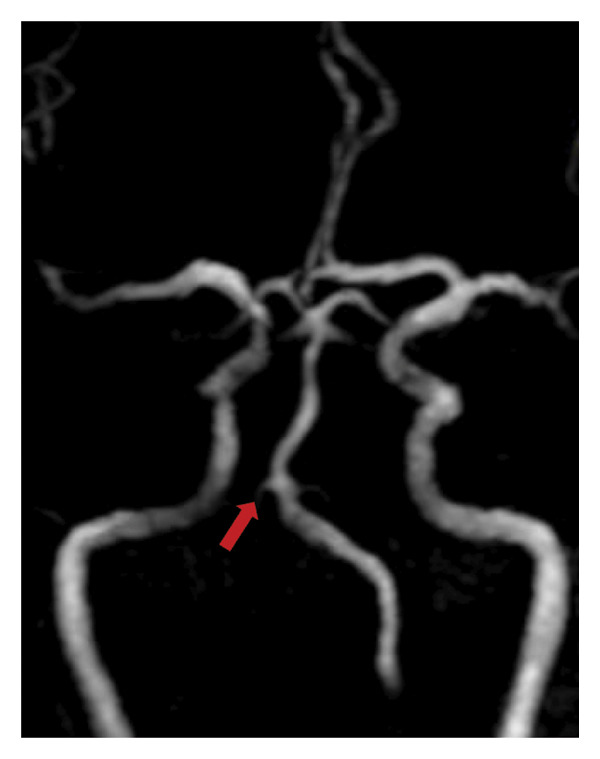
Magnetic resonance angiography (MRA) showing the absence of flow‐related signals in the intracranial segment of the right vertebral artery (red arrow).

Posttracheostomy, the patient was comfortable with a stable sensorium and with no progression of neurologic deficits. BP ranged from 110/60 to 120/70, while capillary blood glucose fluctuated between 160 and 325 mg/dL. Noncontrast magnetic resonance imaging (MRI) of the brain revealed an acute nonhemorrhagic infarct at the bilateral ventromedial aspect of the medulla with the characteristic “heart‐shaped appearance” (Figure 2) in diffusion‐weighted imaging (DWI), indicative of BMMI. Magnetic resonance angiography (MRA) revealed absent flow‐related signals in the right vertebral artery (Figure 3). Secondary stroke prevention with clopidogrel was started, and physical therapy was initiated.

On the fourth day of hospitalization, there was modest improvement in the muscle strength of the right upper and lower limbs, but increased work of breathing and oxygen requirement were observed. Repeat chest radiography and ultrasound revealed progression of pneumonia already involving both lungs, with massive bilateral pleural effusion. On the sixth day of hospitalization, the patient developed intractable septic shock, with a rising procalcitonin level of 11.55 ng/mL, complicated by acute kidney injury, and eventually died from Type 1 respiratory failure secondary to severe pneumonia.

## 3. Discussion

The classic MMI, as proposed by Dejerine, includes the involvement of the pyramid, medial lemniscus, and hypoglossal nucleus, which are all located in the medial aspect of the medulla within the vascular territory of the intracranial segment of the anterior spinal artery (ASA) [4, 5]. Bilateral infarction of these structures produces quadriplegia and lower cranial nerve deficits [3–5]. A 3‐week stepwise progression is atypical for a classic ischemic stroke and may resemble the clinical course of myasthenic crisis or Guillain–Barré syndrome with extensive bulbar involvement. Recognition of subtle yet crucial clinical signs is paramount in promptly diagnosing the condition, even before confirmatory testing. In this case, the presence of bilateral upper motor neuron signs was the pivotal finding that directed our approach toward a central origin of the patient’s symptoms, effectively excluding a neuromuscular junction disorder and an acute polyneuropathy. The coexistence of bulbar manifestations localized the lesion rostral to the spinal cord, thereby excluding a high cervical cord myelopathy. While cervicomedullary involvement in longitudinally extensive transverse myelitis of neuromyelitis optica was compatible with the presenting deficits, onset at this age of the patient argued against this diagnosis. Transient ischemic attack and vertebrobasilar insufficiency were excluded owing to the lack of symptom resolution in the clinical progression, and vertebral artery dissection was deemed less probable in the absence of headache or cervical pain. Basilar artery atherosclerotic disease was a strong differential due to the bilaterality of deficits with a stepwise progression, suggestive of an evolving locked‐in syndrome. However, the complete absence of pontine cranial nerve involvement ultimately supported a medullary localization, leading to our primary working impression of BMMI.

A central rather than peripheral mechanism of vocal cord palsy was favored, as this explanation parsimoniously integrated the patient’s neurological deficits with the neuroimaging findings. Symmetric immobility of the vocal cords on laryngoscopy mirrored the bilateral medullary infarcts seen on brain MRI, implicating the involvement of the nucleus ambiguus or its corticobulbar projections in the medulla. In contrast, peripheral causes typically produce asymmetric involvement of the vocal cords mainly because of the unequal course of the recurrent laryngeal nerves [9]. Thus, further imaging to exclude neoplastic or compressive pathologies along the peripheral pathway was not clinically justified. The converse, however, does not necessarily hold true, as unilateral vocal cord paralysis may still arise from central lesions [9].

**Figure 4:**
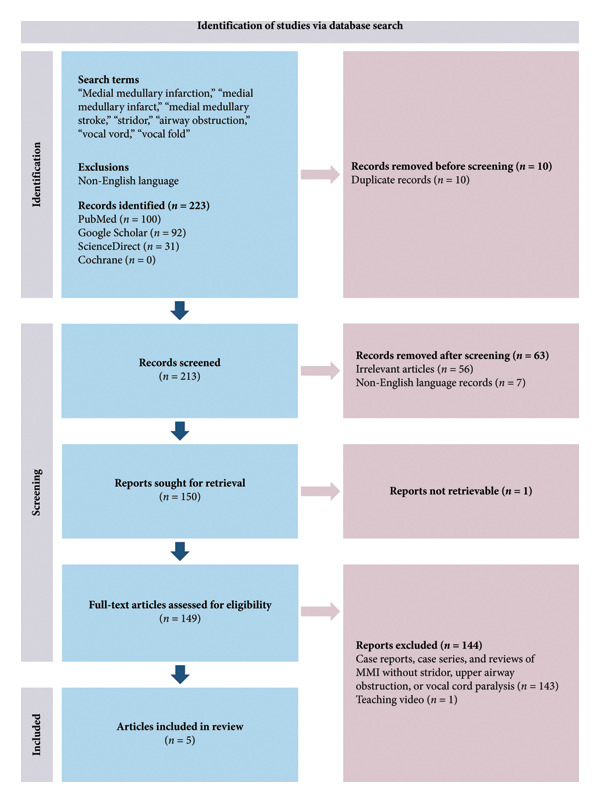
PRISMA flowchart of article identification and screening. LMI, lateral medullary infarction; MMI, medial medullary infarction.

From an anatomical standpoint, the occurrence of vocal cord paralysis in BMMI is theoretically plausible, as the nucleus ambiguus, which contains the motor neurons that innervate the intrinsic muscles of the larynx, is also located in the medulla [10, 11]. Despite this structural proximity, stridor remains a rare manifestation of BMMI. In a prospective study by Kim et al., which included 86 consecutive patients with MMI, 12 of whom had bilateral involvement, and none exhibited signs of upper airway obstruction [12]. Similarly, a systematic review of 38 cases of BMMI by Pongmoragot et al. found no instances of upper airway obstruction or stridor [4]. In our literature review, we conducted a systematic search to identify case reports, series, or reviews of stridor, airway obstruction or vocal cord paralysis in association with BMMI. To maximize sensitivity, the primary search term used was “medial medullary infarction” to capture articles that incorporated both bilateral and unilateral MMI (Figure 4).

Five case reports were identified describing stridor in relation to MMI, and four of these cases were BMMI (Table 1). Three cases had no laryngoscopic evaluation, leaving it unclear whether it was secondary to central vocal cord dysfunction, palatal palsy, or accumulation of secretions. Only the case reported by Tai et al., had BMMI with a laryngoscopically confirmed bilateral vocal cord palsy [8]. Table 1 summarizes the clinical features of previously reported cases of MMI in which stridor was a presenting manifestation.

**Table 1:** Case studies published in the literature of MMI with stridor as a clinical feature.

Case study, author, year	Age, years	Sex	Clinical features	Cranial MRI findings	MRA findings	Laryngoscopy
Tai et al. (2011) [8]	60	M	“Choking sensation,” vomiting, slurred speech, inspiratory stridor, left preferential gaze, left horizontal nystagmus, and asymmetric quadriparesis	Acute bilateral rostral medial medullary infarction affecting the ventral, middle, and dorsal medullary areas	Stenosis of the proximal basilar artery; hypoplastic left vertebral artery; and possible occlusion distal to PICA origin	Bilateral vocal cord palsy

Allam et al. (2014) [13]^†^	47	F	Previous stroke in the right medial medulla 3 months prior Acute nonvertiginous dizziness, loud inspiratory stridor, dysarthria, asymmetric quadriparesis, pseudobulbar affect (current presentation in the case study)	Acute right medial medullary infarction (previous stroke 3 months prior); An acute infarct in the left paramedian pons (current finding in the case study)	—	Bilaterally abducted and bowed in contour, with paradoxical closure on every inspiratory cycle

Paliwal et al. (2009) [14]	70	M	Sudden‐onset vertigo, dysphagia, anarthria, quadriplegia, and inspiratory stridor	Diffusion restriction in the bilateral medial medulla	Hypoplastic right vertebral artery	—

Gallegos Koyner et al. (2023) [15]	39	M	Altered mental status, quadriparesis, poor coordination, and developed inspiratory stridor later in the course of the illness	Acute bilateral medial medullary infarction; acute right subcortical insular infarction; and encephalomalacia of the left pons	Unremarkable	—

Abebe et al. (2025) [16]	70	F	Vertigo, right‐sided facial numbness, dysarthria, quadriparesis, and horizontal nystagmus	Diffusion restriction in the bilateral medial medulla	Occlusion at the distal intradural (V4) segment of the left vertebral artery and multifocal stenosis of the basilar artery^§^	—

*Note:* F, female; M, male.

Abbreviations: MMI, medial medullary infarction; MRA, magnetic resonance angiography; MRI, magnetic resonance imaging.

^†^Unilateral medial medullary infarction.

^§^Computed tomography angiography was performed.

The rarity of bilateral vocal cord paralysis in BMMI may be attributed to the lateral and deep location of the nucleus ambiguus in the rostral medulla, within the vascular territory of the posterior inferior cerebellar artery (PICA) [10]. This may account for the more frequent association of laryngeal dysfunction with lateral medullary infarctions than with MMI [10, 17, 18]. We hypothesize that vulnerability of the nucleus ambiguus in BMMI may depend on the degree of overlap between the ASA and PICA territories within medullary border zones. Arterial territories in the medulla exhibit rostrocaudal variability on clinicotopographic correlation, suggesting the existence of regions of shared perfusion near territory interfaces [19]. In this context, critical ASA hypoperfusion could generate an ischemic penumbra within areas of the PICA territory that ordinarily depend on dual vascular supply for optimal perfusion. Thus, ischemic injury may extend beyond the ASA territory and secondarily involve adjacent structures such as the nucleus ambiguus. The relatively reduced cross‐sectional area in this region of the brain may further accentuate this mechanism, rendering even subtle hemodynamic perturbations clinically significant. Alternatively, the nucleus ambiguus itself may be spared, with laryngeal dysfunction arising from disruption of corticobulbar projections before they synapse onto the nucleus ambiguus [14, 20], rather than from direct nuclear involvement.

Somatotopic organization of the motor neurons in the nucleus ambiguus that innervate the sole vocal cord abductor in the larynx, the posterior cricoarytenoid muscle (PCM), may also provide a neuroanatomical basis for the rarity of airway obstruction in BMMI. Experimental evidence involving rats has elucidated that the PCM receives innervation from motor neurons that are broadly distributed along the central rostrocaudal regions of the nucleus ambiguus [21]. This may suggest that, in order to produce paralysis of the vocal cords in the adducted median–paramedian position severe enough to cause upper airway obstruction, an extensive involvement of the nucleus ambiguus may be necessary to completely impair the vocal cord abductor. However, this observation is derived primarily from animal studies, and corroborative data in humans remain limited.

Vocal cord paralysis secondary to involvement of the nucleus ambiguus or its corticobulbar projections is likewise expected to have concomitant impairment of deglutition, palatal function, and cough reflex, given their shared nuclear origin of innervation [11]. If left unaddressed, the dysfunction increases the risk of aspiration pneumonia. The delayed arrival at the ED likely allowed for the development of this complication, supported by chest radiographic findings, and elevated procalcitonin levels upon arrival at the ED. This is further compounded by the poorly controlled diabetes mellitus, increasing her vulnerability to infection that has accelerated the progression to sepsis, parapneumonic effusion, and refractory shock, ultimately contributing to her demise.

Absent flow‐related signals in the right vertebral artery on MRA may have reflected occlusion, stenosis, or hypoplasia. Basiparallel anatomical scanning or CT angiography would have ideally distinguished among these possibilities; however, these modalities were no longer performed, representing a limitation in identifying the exact vessel responsible for causing BMMI in this case. Two possible mechanisms remain. First, if the right vertebral artery was merely hypoplastic, the occlusion or stenosis may have been in the ASA itself. Second, if the patient had a true occlusion or stenosis of the right vertebral artery, BMMI arising from the involvement of a single vertebral artery may be attributed to anatomical variations in the origin of the ASA, which occur in approximately 10% of individuals [2]. In the microanatomical study of Er et al., three major ASA origin types were described, with Type II characterized by a solitary ASA arising from either the right or left ramus [22]. In this variant, occlusion of the vertebral artery distal to the origin of the PICA and proximal to the ASA trunk may result in isolated ischemia of medial medullary structures producing BMMI.

## 4. Conclusion

This case highlights the importance of recognizing the rare association between BMMI and signs of upper airway obstruction, an unusually complex presentation that may confound the diagnostic process. Precise neurological examination, with neuroanatomical correlation, is indispensable in differentiating this entity from other causes of vocal cord paralysis. The variable overlap in medullary arterial territories and possible corticobulbar involvement provide plausible explanations for the occurrence of this manifestation in BMMI. Further, the concurrent BMMI resulting from a single vertebral artery compromise underscores the significance of recognizing anatomical variants in the intricate neurovascular relationships within the medulla. This nuanced understanding is critical for accurate diagnosis and timely management, emphasizing the need for heightened vigilance among clinicians when confronted with complex neurological presentations.

## Ethics Statement

In accordance with the institutional review board policies, the need for approval was waived for this study owing to its nature as a case report involving fewer than five patients. Written and verbal consent from the next of kin was obtained in the writing of this case report.

## Consent

Please see the Ethics Statement.

## Conflicts of Interest

The authors declare no conflicts of interest.

## Funding

The authors did not receive any funding to fulfill this report.

## Data Availability

The data that support the findings of this study are available upon request from the corresponding author. The data are not publicly available due to privacy or ethical restrictions.
